# Indents

**Published:** 1901-06-15

**Authors:** 


### INDENTS.

**☞ Brief Articles of Technical or Scientific Value will receive notice and comment. Contributions invited.**

TOOTHACHE WAX.—Hard paraffin, 1 dr.; Burgundy pitch, 1 dr.; oil of cloves, 20 mins.; creosote, 20 mins. Melt together the first two ingredients, and when they are nearly cool add the other two, and make the mass which is thus formed into pills or small cones.—*Pacific Drug Review.*

COMBINATION FILLINGS OF CEMENT AND AMALGAM.—Dr. Strang prepares his amalgam as usual ready for filling, then places it in a mortar and adds to it a quantity of cement powder equal to one-third of its volume, and thoroughly mixes the two together. He then adds the cement liquid and mixes as for an ordinary oxyphosphate filling to the consistency of hard putty, and inserts it at once into the cavity, to which it readily adheres.—*Cosmos.*

CROWNS FOR FIRST MOLARS IN CHILD OF TEN.—A correspondent asks for advice that perhaps some of our readers will be pleased to give: "Would you advise crowning first molars at the age of ten? I have a little girl of ten whose molars are decaying very rapidly, such as a soft chalky ring completely around the tooth, as well as defective pits. In no case is the decay deep enough to necessitate devitalization. Both parents have a decided tendency to pyorrhea, which might cause the crowns to be intolerable to her gums."—*Dominion Dental*

*Journal.* [The first molar is the most important tooth in the entire denture, and is to be saved in every case where it is at all practicable.—Ed.]

Cavity Preparation.—"In most cases I should prepare my cavities in accordance with the following general rules: Teeth of good structure, in a healthy mouth, having small cavities, may be filled without cutting till the margins are free. Teeth of medium quality should be cut till the margins of the cavities are just free on the buccal and lingual sides, and if not cut through from the grinding surface, should be so cut as to allow the gold to come just in sight along the proximo-grinding surface borders, after the teeth have closed. Then the only danger exists at the cervical margin. In very frail teeth some small cavities may not be safe until cut up to, and sometimes even under the gum."—Dr. S. G. Perry, in *Items of Interest.*

Advisability of Administering Anesthetics.—The question as to the advisability of resorting to general anesthesia in dental practice must be settled by each person according to his best judgment, and in relation to the special case in hand. Broadly speaking, it would not seem justifiable to take the risk of administering chloroform or ether for an ordinary extraction, while it might be absolutely necessary in a case of an impacted tooth, or the excision and removal of root ends in treatment of alveolar abscesses. It should be remembered, however, that anesthetics, as administered by physicians, are handled usually quite differently from the methods employed by dentists.

It would seem wise for the dentist to resort to general anesthesia only when driven to it by necessity, and then if it be impossible to have a medical man as the anesthetizer to assist him during the operation, he should take command of the case with the authority and confidence of the regular surgeon, and should not hesitate to have his patient sufficiently freed from tight garments and in the proper position to reduce the risk to a minimum.—Editorial in *Items of Interest*.

Use of Gutta-Percha in Exposing Margins.—When caries attack the proximal surface of a tooth it usually leaves the gingival margin of the cavity curved with its convexity looking toward the alveolar process, and if the gutta-percha is merely packed into the cavity and across the interproximal space against the adjacent tooth it leaves the buccal (or labial) and lingual festoons of gum standing farther crownwise than the gum midway between the teeth. These festoons hold the rubber dam from readily passing far enough rootwise to expose the gingival margin of the cavity, and they are also in the way of finishing strips or files and are likely to be lacerated. To prevent this the gutta-percha should be made to extend laterally each way so as to force the festoons of gum back on a level with the gum between the teeth. This will result in such an exposure of the space that a perfect view is had of the entire operation and ready access is gained for the insertion and finishing of the filling. When the gum is treated in this way, it will very rapidly reoccupy the interproximal space and cover the gingival portion of the filling after the operation.—C. N. Johnson, in *Items of Interest*.

